# Special Issue: “Bacteriophages and Biofilms 2.0”

**DOI:** 10.3390/v18010049

**Published:** 2025-12-29

**Authors:** Zuzanna Drulis-Kawa, Tomasz Olszak

**Affiliations:** Department of Pathogen Biology and Immunology, University of Wroclaw, 51-148 Wroclaw, Poland

The natural ability of certain bacterial species to form biofilms presents numerous challenges for modern medicine and the food and pharmaceutical industries. The multi-layered structure of biofilm, containing cells with diverse phenotypes and metabolic activity, is embedded in a matrix composed of exopolysaccharides (EPSs), eDNA, and proteins, which hinders the eradication of such bacterial consortia from both biotic and abiotic surfaces. Biofilm is a kind of global virulence factor that drastically reduces the effectiveness of classic antibacterial agents (antibiotics, chemotherapeutics, and disinfectants) as well as components of the immune system.

Bacteriophages, as an integral part of bacterial life and evolution, affect biofilms in various ways. On the one hand, temperate phages (or domesticated prophages) are often necessary for biofilm formation (they are responsible for adhesion and microcolony formation; e.g., filamentous phage Pf4 in *Pseudomonas aeruginosa*). The induction of such prophages or the expression of individual viral genes is controlled by bacterial quorum-sensing (QS) signalling systems (e.g., in *Vibrio anguillarum* phage H20). Conversely, prophages may also influence the mechanisms of bacterial intercellular communication (e.g., Pf phage in *P. aerugonosa* or phage ARM81ld in *Aeromonas* sp.). On the other hand, lytic phages, armed with enzymes that degrade the biofilm matrix (e.g., depolymerases) or lysins effective against both planktonic and dormant cells, can become an attractive tool in combating persistent colonisation by biofilm-forming bacterial strains.

This Special Issue of *Viruses*, entitled ‘Bacteriophages and Biofilms 2.0’, contains 10 original research papers and 2 comprehensive reviews that bring science closer to a better understanding of the importance of phages regarding the formation, maintenance, and dispersion of biofilms. The topics of the selected articles focus on three issues: the interaction of phages with bacterial biofilms, the therapeutic use of phages against multidrug-resistant (MDR) pathogens, and the use of phages in food preservation.

Naturally occurring bacterial biofilms exhibit different characteristics and dynamics compared to those cultivated in laboratories (in vitro). Two research groups featured in this Special Issue emphasised the importance of the environment in which a given species of bacteria exists and the impact of this environment on the biofilm formation process and biofilm–phage interactions.

Guła et al. [1] demonstrated that, surprisingly, exposure to phages specific to *Klebsiella pneumoniae* stimulates biofilm formation in *P. aeruginosa*. This suggests the involvement of unspecified regulatory mechanisms that detect the presence of phages (not only species-specific ones). It suggests also that bacteria are able to sense phage virions, regardless of specificity, triggering biofilm matrix formation, blocking potential phage adsorption and infection in advance.

The work of Li et al. [2] shows how high salt concentrations stimulate the VP3 phage to lyse the biofilm cells of *Vibrio cholerae*. The combination of salt and phages increases penetration and destabilises the biofilm matrix, leading to more effective elimination of bacteria. These findings may contribute to improving the control of environmental reservoirs of *V. cholerae* and preventing seasonal cholera outbreaks.

An engaging review by Bucher & Czyż [3] discusses the mechanism of superinfection exclusion (SIE), in which prophages modify the host to block future phage infections. The authors describe the impact of SIE on motility, adhesion, biofilm production, conjugation, and resistance, as well as the implications for phage therapy. The paper emphasises that SIE may reduce the effectiveness of phage cocktails and requires circumvention strategies (e.g., selection of phages insensitive to SIE). Links to bacterial signalling networks and the ecology of multispecies biofilms are also discussed.

Combating infections caused by multidrug-resistant bacterial strains is one of the greatest challenges facing medicine, which has been searching for alternative antibacterial therapies for years. Therefore, the search for new phages and their thorough characterisation is a crucial part of preparing effective phage-based medicine.

Soro et al. [4] describe the isolation and complete characterisation of the lytic phage vB_Efs8_KEN04, active against clinical MDR strains of *Enterococcus faecalis* prevalent in Kenya. The authors present a wide range of antimicrobial activity and the ability to reduce biofilms in vitro. Genomic analysis did not reveal any genes associated with toxicity or drug resistance, which increases the therapeutic potential of vB_Efs8_KEN04 phage.

Plumet et al. [5] isolated and characterised six new phages (SAVMO1–SAVMO6) specific for clinical isolates of *Staphylococcus* sp. associated with diabetic foot ulcers (DFUs). The morphology, host range, and genomic profiles, indicating the absence of toxic genes, were presented. Isolated phages effectively reduced biofilms under laboratory conditions, which may support the treatment of difficult-to-heal wounds. The authors propose formulating phage cocktails for greater strain coverage.

Obradović et al. [6] describe the isolation of two new phages (LASTA and SJM3) active against MDR strains of *K. pneumoniae*. The authors examined their narrow host range, stability, and genomic structure. The mechanisms of host resistance development and the consequences for the sustainability of phage therapy were also analysed. The results support the design of cocktails and sequential administration strategies to limit the selection of resistant variants. The paper highlights the importance of monitoring the evolution of resistance during the treatment of *Klebsiella* biofilm infections.

Peters et al. [7] characterised two virulent T4-like phages infectious to *Acinetobacter baumannii*. Morphological, genomic, and host profile data confirming therapeutic potential were presented. In in vitro models, myoviruses DLP1 and DLP2 showed strong lytic activity of and biofilm reduction in *A. baumannii*. Safety analyses did not reveal any undesirable genes, which favours potential clinical use. The study supports the development of phage cocktails for multidrug-resistant *A. baumannii* infections.

The search for alternative antimicrobial therapies does not mean giving up on antibiotics. In many cases, the use of phages and antibiotics can have synergistic effects.

The study by Joo et al. [8] evaluates the effectiveness of combining phages and antibiotics against *S. aureus* biofilms on implant materials. Under static and flow conditions, the combinations were more effective than monotherapies. The results indicate synergistic mechanisms, including increased phage penetration and weakening of the biofilm matrix by antibiotics. The authors suggest the use of integrated protocols in the prevention and treatment of implant-related infections.

Research conducted by Alipour-Khezri [9] examines the synergistic effect of coupling phages with green-synthesised zinc oxide (ZnO) nanoparticles against *P. aeruginosa*. The authors demonstrated a significant reduction in biofilm density and cell viability for combination therapy compared to each agent used separately. The mechanism involves both the lytic activity of the phage and damage to the EPS matrix structure caused by the ZnO. The study highlights the safety and potential application of this solution in medicine. The authors suggest that integrated nano-phage strategies may limit the development of resistance.

Phage therapy using intact virions has certain limitations. In parallel with research on phages themselves, many research teams are focusing on the use of recombinant phage enzymes.

Wesołowski et al. [10] prepared a review focusing on the diversity of phage endolysins potentially active against uropathogenic *E. coli* (UPEC) strains. In silico and phylogenetic analyses show diversity in length, mass, isoelectric point, and functional domains. The authors discuss protein architecture, stability, and modification options to increase anti-biofilm activity. The review highlights the potential of endolysins as an alternative or supplement to antibiotics in urinary tract infections.

Bacterial contamination and biofilms are not only a medical problem. The pharmaceutical and food industries can also face issues such as decreased process efficiency or product degradation caused by microbes. In such situations, phages can also be used, both in a preventive and interventional context.

The paper by Hille et al. [11] describes the filamentous phage PMBT54 infecting milk spoilage bacteria: *P. carnis* and *P. lactis*. Filamentous phages usually do not cause lysis but are secreted from cells, and can modulate the host phenotype, including biofilm formation. The study presents taxonomic, genomic, and phenotypic characteristics of phages classified to *Tubulavirales* order. The results are relevant for food spoilage control and surface biocontrol in the dairy industry.

Jin et al. [12] present phage vB_EfaS_WH1 isolated from chicken faeces, active against *E. faecalis* biofilms. Effective biofilm removal and growth inhibition on poultry meat surfaces were demonstrated. Phylogenetic and microscopic analysis confirmed the virulent nature and stability of the phage. The results indicate applications in food safety as a biocontrol of pathogens. The study suggests that rinsing meat with phage preparations may reduce the risk of contamination and the occurrence of bacterial antibiotic resistance in the food chain.
